# Neuroprotective Effects of dl-3-n-Butylphthalide against Doxorubicin-Induced Neuroinflammation, Oxidative Stress, Endoplasmic Reticulum Stress, and Behavioral Changes

**DOI:** 10.1155/2018/9125601

**Published:** 2018-08-16

**Authors:** Dehua Liao, Daxiong Xiang, Ruili Dang, Pengfei Xu, Jiemin Wang, Wenxiu Han, Yingzhou Fu, Dunwu Yao, Lizhi Cao, Pei Jiang

**Affiliations:** ^1^Department of Pharmacy, Hunan Cancer Hospital, Changsha 410011, China; ^2^Institute of Clinical Pharmacy & Pharmacology, Second Xiangya Hospital, Central South University, Changsha 410011, China; ^3^Department of Pharmacy, Jining First People's Hospital, Jining Medical University, Jining 272000, China

## Abstract

Doxorubicin (DOX) is a broad-spectrum antitumor drug while its use is limited due to its neurobiological side effects associated with depression. We investigated the neuroprotective efficacy of dl-3-n-butylphthalide (dl-NBP) against DOX-induced anxiety- and depression-like behaviors in rats. dl-NBP was given (30 mg/kg) daily by gavage over three weeks starting seven days before DOX administration. Elevated plus maze (EPM) test, forced swimming test (FST), and sucrose preference test (SPT) were performed to assess anxiety- and depression-like behaviors. Our study showed that the supplementation of dl-NBP significantly mitigated the behavioral changes induced by DOX. To further explore the mechanism of neuroprotection induced by dl-NBP, several biomarkers including oxidative stress markers, endoplasmic reticulum (ER) stress markers, and neuroinflammatory cytokines in the hippocampus were quantified. The results showed that dl-NBP treatment alleviated DOX-induced neural apoptosis. Meanwhile, DOX-induced oxidative stress and ER stress in the hippocampus were significantly ameliorated in dl-NBP pretreatment group. Our study found that dl-NBP alleviated the upregulation of malondialdehyde (MDA), nitric oxide (NO), CHOP, glucose-regulated protein 78 kD (GRP-78), and caspase-12 and increased the levels of reduced glutathione (GSH) and activities of catalase (CAT), glutathione reductase (GR), and glutathione peroxidase (GPx) in the hippocampus of rats exposed to DOX. Additionally, the gene expression of interleukin-6 (IL-6), interleukin-1*β* (IL-1*β*), and tumor necrosis factor-alpha (TNF-*α*) and protein levels of inducible nitric oxide synthase (iNOS) were significantly increased in DOX-treated group, whereas DOX-induced neuroinflammation was significantly attenuated in dl-NBP supplementation group. In conclusion, dl-NBP could alleviate DOX-induced anxiety- and depression-like behaviors via attenuating oxidative stress, ER stress, inflammatory reaction, and neural apoptosis, providing a basis as a therapeutic potential against DOX-induced neurotoxicity.

## 1. Introduction

Doxorubicin (DOX) is an anthracycline antibiotic used commonly in multidrug chemotherapy regimens to treat solid tumors [1, 2]. However, its use as a drug has been reported to cause some adverse effects like heart arrhythmias, neutropenia, cardiotoxicity, kidney injury, as well as neuron damage in the brain [1, 3, 4]. Notwithstanding the fact that DOX poorly crosses the blood-brain barrier, it still penetrates the brain at doses sufficient to cause cytotoxicity [5]. More and more evidence showed that neurotoxicity is accompanied with long-term use of DOX and may cause many neuropsychiatric diseases including depression, anxiety, and impaired cognitive function [6, 7]. Clinical study also showed that DOX treatment has a negative impact on cognitive function in women with breast cancer [8].

Several studies suggested that the pathogenesis of anxiety and depression is associated with oxidative stress and neuroinflammatory response particularly in the hippocampal region [9–11]. Hydroxyl radicals and superoxide radicals along with hydrogen peroxide are produced after administration of DOX, leading to the alterations of oxidative stress and antioxidant defense system. It is assumed that the formation of free radicals induces oxidative stress and plays a crucial role in the mechanism of DOX-induced neurotoxicity [12, 13]. Moreover, the generation of superoxide anions induced by DOX can elevate the level of circulating necrosis factor-alpha (TNF-*α*) which can directly cross the blood-brain barrier and activate glial cells to initiate the local production of proinflammatory cytokines, exacerbating oxidative stress and neural apoptosis [14]. In addition, DOX-evoked reactive oxygen species (ROS) activate nuclear factor kappa B (NF-*κ*B) signaling pathway, which in turn triggers the activation of proinflammatory cytokines, such as interleukin-6 (IL-6), interleukin-1*β* (IL-1*β*), and TNF-*α* [11], and inducible nitric oxide synthase (iNOS) expression. Moreover, DOX specifically activates part of the endoplasmic reticulum (ER) stress response pathway, thus contributing to its proinflammatory effect in the hippocampus [15]. These inflammatory mediators have been shown to be involved in neuroinflammation both in animal models and patients undergoing chemotherapy [16]. The resulting neuroinflammation can trigger apoptotic cell death and depletion of neurotrophic factors, causing neurobehavioral alterations [11, 17]. Besides, previous study showed that ER stress and disrupted neurogenesis in the brain are associated with cognitive impairment and depression-like behavior in rats following chronic stress exposure [18]. Cai et al. have also reported that ER stress plays an important role in potential memory impairments in rats treated with microcystin leucine arginine [19]. Therefore, it might be beneficial to reduce the production of ROS and inhibit the release of neurotoxic agents in the treatment of DOX-induced depression.

Recent studies have highlighted that dl-3-n-butylphthalide (dl-NBP) displays an important role in mitigating brain damage [20, 21]. dl-NBP is a synthesized compound based on l-3-n-butylphthalide which is extracted from the seeds of *Apium graveolens* Linn. dl-NBP had been approved by the State Food and Drug Administration of China for clinical use in patients with stroke in 2002 [22]. As a fat-soluble small molecule compound, dl-NBP could cross the blood-brain barrier efficiently. dl-NBP has a variety of protective effects on brain tissues as a multitarget drug. Previous study has found that dl-NBP reduces focal cerebral ischemia volume in rats and improves local cerebral ischemia brain-induced edema, brain energy metabolism disorder, and apoptotic neuronal cell death [21]. Several studies have reported the neuroprotective effects of dl-NBP, for example, dl-NBP enhanced the ability of learning and memory in animal models of Alzheimer's disease and provided neuroprotection in the mice models after traumatic brain injury [23, 24]. It has also been reported that dl-NBP relieves hypoxia-induced damage in vitro [20] and prolonged animal survival in the mouse model of amyotrophic lateral sclerosis [24]. In addition, studies also showed that dl-NBP could resist HSPB8 K141N mutation-induced oxidative stress [22] and attenuate amyloid-*β*-induced inflammatory responses in cultured astrocytes [25, 26], highlighting the neuroprotective effect of dl-NBP via alleviating oxidative stress and inflammatory responses.

Based on the above findings, the purpose of our study was to investigate the potential protective effects of dl-NBP against DOX-induced neurotoxicity and depression-like behaviors in rats. In addition, the possible underlying mechanisms, including antioxidant, anti-inflammatory, and anti-ER stress as well as antiapoptotic effects of dl-NBP, were also examined.

## 2. Materials and Methods

### 2.1. Animals

Sprague-Dawley rats (male, 180–220 g; the Experimental Animal Center of Hunan Cancer Hospital) were initially housed in groups in a temperature-controlled (23 ± 2°C) environment under a 12/12 h light/dark cycle with free access to food and water, prior to sucrose preference test (SPT). This study was approved by the Animal Care and Use Committee of Hunan Cancer Hospital (protocol number 027/2016). All experiments were performed in accordance with the Guide for Care and Use of Laboratory Animals (Chinese Council).

### 2.2. Experimental Design

Animals were divided randomly into three groups (*n* = 8): (1) control, (2) DOX, and (3) DOX + dl-NBP. The untreated control group was injected with 1.5 ml of normal saline. Rats in the DOX group were given DOX every two days for a total of seven injections via intraperitoneal injection at a dose of 2.5 mg/kg for each injection. The dose and treatment duration was chosen based on our previous research showing the DOX-induced neurotoxicity and depression-like behaviors [27]. The DOX + dl-NBP group received dl-NBP (30 mg/kg) daily by gavage for three weeks starting one week before giving DOX. The dose of dl-NBP was selected because of previous investigation showing neuroprotective effects of this drug against cerebral ischemia and brain injury [22]. In addition, dl-NBP was administrated one week before DOX treatment to fully activate the antioxidant system, protecting the brain against the relative high dose of DOX challenge. The body weight of these rats was monitored throughout the experiment, and drug doses were adjusted accordingly.

At the end of the three weeks, behavioral tests were carried out following the sequence of SPT, elevated plus maze (EPM) test, and forced swimming test (FST). After behavioral tests, the rats were anesthetized with sodium pentobarbital (50 mg/kg) via intraperitoneal injection [28]. Blood samples were taken from cardiac coronary artery after anesthesia, and the brains were quickly removed after cardiac perfusion with phosphate-buffered saline (PBS) (pH = 7.2). The left hemisphere of the brain was maintained in 4% paraformaldehyde and then embedded in paraffin, prepared for histopathological examination and immunohistochemical staining. For the right hemisphere, the hippocampus was dissected and used for oxidative stress measurement and Western blot and polymerase chain reaction (PCR) analysis. The biochemical parameters such as malondialdehyde (MDA), nitric oxide (NO), reduced glutathione (GSH), glutathione peroxidase (GPx), glutathione reductase (GR), catalase (CAT), IL-1*β*, IL-6, TNF-*α*, glucose-regulated protein 78 kD (GRP-78), iNOS, p65, inhibitor of NF-*κ*B (I*κ*B), CHOP, and caspase-12 were determined in our study.

### 2.3. Behavioral Test

#### 2.3.1. Elevated Plus Maze Test

EPM test was performed to assess the anxiety-like behavior in rats. The apparatus of EPM was consisted of two open arms (35 × 5 cm^2^) which were perpendicular to two closed arms (35 × 5 cm^2^) with a small central square (5 × 5 cm^2^) between arms. The maze was elevated 50 cm from the floor in a dimly illuminated room. Each rat was placed at the center of maze with head facing towards the open arm and allowed to freely explore for 5 min. The total number of entries into the open arm, closed arm, and time spent in open arm during the test were recorded and evaluated [29].

#### 2.3.2. Sucrose Preference Test

SPT was utilized to determine anhedonia response which is a core symptom of major depression in rats. Prior to SPT, all the rats were housed individually and habituated to 48 h of forced 1% sucrose solution consumption in two bottles on each side. Then after 14 h of water deprivation, the rats were given access to two preweighed bottles, one containing 1% sucrose solution and another containing tap water. The position of the bottles was alternated to avoid bias from place preference. The bottles were weighed again after 1 h, and the weight difference was considered to be the rat intake from each bottle. The preference for sucrose was measured as a percentage of the consumed 1% sucrose solution relative to the total amount of liquid intake [30, 31].

#### 2.3.3. Forced Swimming Test

Antidepressant efficacy and depression-like behavior in rodents were screened by using FST. The test was performed as previously described with minor modifications [28]. In brief, each rat was placed in a plastic cylinder (45 cm height and 25 cm diameter) containing approximately 35 cm of water (24 ± 1°C) for a 15 min pretest. After swimming, rats were dried with towels and placed back in their home cage. Twenty-four hours later, the rats were exposed to the same experimental conditions outlined above for a 5 min FST, and immobility time was recorded in our study.

### 2.4. Biochemical Parameter Assay

A 10% (*w*/*v*) homogenate of the hippocampus in 0.1 M phosphate-buffered saline (PBS) at pH 7.4 was prepared and centrifuged at 9500 rpm for 20 min at 4°C. The supernatant was used for the measurement of biochemical parameters of MDA, NO, GSH, GPx, and CAT.

#### 2.4.1. Measurements of MDA and NO Content

The MDA formation was spectrophotometrically measured by the thiobarbituric acid (TBA) reaction [32]. Briefly, 1 ml of 15% trichloroacetic acid was added to 500 *μ*l of brain homogenate supernatant and mixed well, and then, the solutions were centrifuged at 3000 rpm for 10 min. One milliliter of the supernatant was added to 0.5 ml of 0.7% TBA, and then, the mixture was heated for 60 min at 90°C. The absorbance was recorded at 532 nm using UV spectrophotometer. The content of NO was determined according to Montgomery and Dymock's method [33]. The reddish-purple azo dye product was measured spectrophotometrically at 540 nm.

#### 2.4.2. Determination of Antioxidant Parameters

The content of reduced GSH was determined by the method of Maris [34]. GSH was reacted with 5,5′-dithio-bis-2-nitrobenzoic acid generating a yellow chromophore, and the absorbance was measured at 412 nm using a UV spectrophotometer. Total GSH content was expressed as nmol/mg protein.

The CAT activity was measured in brain homogenate following the method of Sinha [35]. A decrease in absorbance due to H_2_O_2_ degradation was monitored at 240 nm for 1 min, and the enzyme activity was expressed as U/mg protein.

The GR activity was assessed according to the previously described method, which was determined by measuring the rate of NADPH oxidation at 340 nm due to the formation of GSH, from GSSG, by the action of GR present in the sample [36]. The unit of enzyme activity was expressed as U/mg protein.

The GPx activity was analyzed as described previously, which was measured by a spectrophotometric method based on the disappearance of NADPH [37]. GPx catalyzes the oxidation of GSH by cumene hydroperoxide. The GPx activity was determined by subtracting the excess GSH after the enzymatic reaction of the total GSH in the absence of the enzyme. GSH reacts with DTNB to form a yellow-colored chromophore which was measured with a spectrophotometer at 412 nm. The enzyme activity was expressed as U/mg protein.

### 2.5. Western Blot Analysis

For Western blot analysis, total protein was prepared from the hippocampus, and the concentration was determined using Bradford method [27]. In brief, the hippocampus sample was loaded on a precast 12% SDS-PAGE gel with 10 *μ*g proteins in each lane. Proteins in the gels were transferred to a 0.45 *μ*m PVDF membrane at 15 V for 15 min in a transfer buffer, pH 8.1 (47.8 mM Tris/HCl, 0.293% glycine, 20% methanol) and blocked for 1 h in 5% nonfat dry milk in TBS-T (25 mM Tris, pH 7.5, 150 mM NaCl, 0.05% Tween-20). The membranes were probed overnight at 4°C with primary antibodies as follows: anti-iNOS (Proteintech; 1 : 500), anti-I*κ*B (Cell Signaling; 1 : 1000), anti-p65 (Proteintech; 1 : 800), anti-GRP78 (Proteintech; 1 : 1000), anti-CHOP (Cell Signaling; 1 : 1000), anti-caspase-12 (Proteintech; 1 : 1000), and anti-*β*-actin (Proteintech; 1 : 4000). After that, the membranes were washed and probed with HRP-conjugated secondary antibody for 40 min at room temperature. The bound antibodies were visualized using an enhanced chemiluminescent detection system (Amersham Pharmacia Biotech, Piscataway, NJ, USA) and then exposed to X-ray films (Kodak Xomat, Rochester, NY, USA). The density of protein bands was quantified using ImageJ software (National Institutes of Health, Bethesda, MD, USA). The density ratio represented the relative intensity of each band against *β*-actin as the loading control and normalized to those in the control group.

### 2.6. Real-Time PCR Analysis

The mRNA levels of inflammatory factors IL-1*β*, IL-6, and TNF-*α* were quantified by quantitative real-time polymerase chain reaction. The reference sample for the study was dissected from the rats in the control group. Total RNA was extracted from the hippocampus using TRIzol reagent (Invitrogen Corp., Carlsbad, CA, USA). The RNA concentration was determined for quantity by using the spectrophotometry (Jingke, Ningbo, China). Complementary DNA (cDNA) was generated from 2 *μ*g of total RNA by RevertAid First Strand cDNA Synthesis Kit (Thermo Fisher Scientific, Tewksbury, MA, USA) using oligo (dT)12-18 as a primer in a total volume of 20 *μ*l. Quantitative PCR was performed on Bio-Rad Cx96 Detection System (Bio-Rad, Hercules, CA, USA) using SYBR green PCR kit (Applied Biosystems Inc., Woburn, MA, USA) and gene-specific primers. The primer sequences were selected according to our previous study [27], and the sequences of gene-specific primers are listed in Table 1. The PCR amplification program consisted of a preincubation at 95°C for 10 min to activate the FastStart Taq DNA polymerase, followed by 40 cycles of denaturation at 95°C for 15 s and a 30 s annealing and elongation step at 60°C. After the amplification procedure, subject all PCR reactions to a melting curve analysis with continuous fluorescence measurement from 65°C to 95°C. Typically, collect one data point in each cycle by a stepwise increase of the temperature by 0.5°C per cycle. Melting curve analysis showed the single and sharp transition, indicating the specificity of the amplifications (Figure S1). The signals were normalized to *β*-actin as an internal standard. All PCR experiments were performed in triplicate. Relative change in mRNA expression was evaluated by using the 2^−ΔΔCq^ method.

### 2.7. Histopathological Examination

For each rat, brain samples were collected and fixed in 4% paraformaldehyde in PBS (pH 7.2) at room temperature overnight and processed routinely for embedding in paraffin. The paraffin-embedded tissue sections (5 mm) were stained with hematoxylin and eosin using standard techniques, and then examination was done through the light electric microscope (Olympus, USA).

### 2.8. TUNEL Assay

Terminal deoxynucleotidyl transferase-mediated deoxyuridine triphosphate nick end labeling (TUNEL) assay was used to evaluate neurocyte apoptosis. The TUNEL method was employed using an apoptosis detection kit (KeyGen Biotech, Nanjing, China). TUNEL-positive tubular cell numbers were counted at random in 20 nonoverlapping cortical fields under 200x magnification. This ratio represented the apoptotic index of the sample and was compared between groups.

### 2.9. Immunohistochemical Staining

For immunohistochemical, hippocampus sections were incubated overnight with anti-iNOS antibody (Santa Cruz Biotechnology, 1 : 500 in PBS, *v*/*v*). Sections were then washed with PBS and incubated with secondary antibodies. For quantitative analysis, original immunohistochemical photographs were assessed by densitometer using MacBiophotonics ImageJ 1.41a software [38, 39].

### 2.10. Statistical Analysis

In this study, all data were analyzed using the Statistical Package for Social Science (SPSS) version 18 (SPSS Inc., Chicago, IL, USA). All brain parameters were presented as means ± SEM and analyzed statistically by one-way analysis of variance (ANOVA) with least significant difference (LSD) post hoc multiple comparisons. The prior level of significance was established at *p* < 0.05.

## 3. Results

### 3.1. Effects of dl-NBP Pretreatment on DOX-Induced Body Weight Gain and Anxiety- and Depression-Like Behaviors

**Figure 1:**
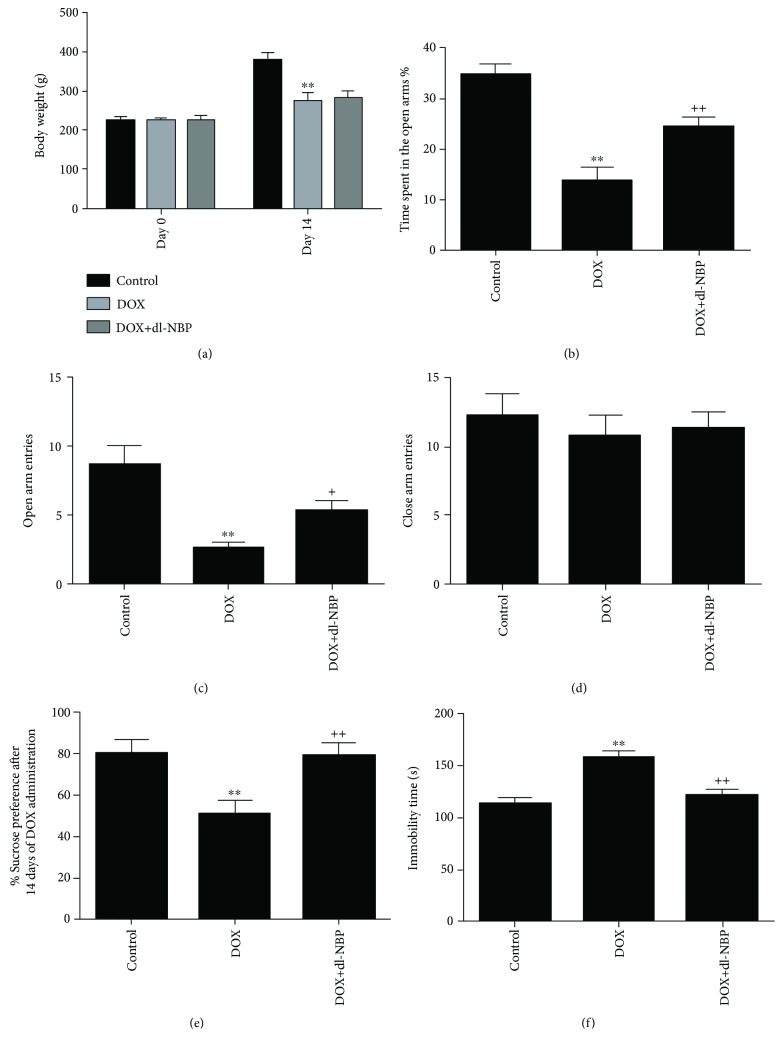
Body weight gain and behavioral test. Effects of DOX and dl-NBP on body weight gain (a); EPM test: time spent in the open arms (b); open arm entries (c); closed time entries (d); SPT: sucrose preference (e); and FST: immobility time (f). Data are expressed as means ± SEM (*n* = 8). ^∗∗^ *p* < 0.01 compared to the control group. ^+^ *p* < 0.05 and ^++^ *p* < 0.01 compared to the DOX-injected group.

As shown in Figure 1(a), DOX-treated rats showed significant decreases in body weight gain when compared to the control animals, whereas dl-NBP pretreatment had no influence on the body weight gain in DOX-treated rats, which was consistent with the results of previous studies [40, 41]. EPM tests were performed for anxiety-like behavior assessment. As shown in Figures 1(b)–1(d), DOX administration induced an anxious effect as evident by reduction of time spent in the open arms (*F*_2,21_ = 18.25; *p* < 0.01) and number of entries in the open arm (*F*_2,21_ = 16.32; *p* < 0.01) as compared to the normal control group. Number of entries (*p* < 0.01) and time spent in the open arms (*p* < 0.01) were significantly increased in the dl-NBP-pretreated group when compared with DOX-treated group. There was no significant difference concerning the parameters of closed arm entries in the three groups.

Depression-like behaviors were assessed by using SPT and FST. In SPT, DOX-exposed rats showed a significant reduction in the sucrose preference (*F*_2,21_ = 9.64; *p* < 0.01) as compared to the normal control group, indicating depression-like behavior caused by DOX exposure (Figure 1(e)). However, dl-NBP pretreatment significantly alleviated DOX-induced depression-like behavior (*F*_2,21_ = 9.64; *p* < 0.01) as indicated by the marked increase in sucrose preference.  Figure 1(f) depicts that immobility time in FST was significantly increased after administration of DOX (*F*_2,21_ = 10.35; *p* < 0.01) when compared with the control group, which was also alleviated by dl-NBP treatment.

### 3.2. Effects of DOX and dl-NBP on Oxidative Stress Markers

**Figure 2:**
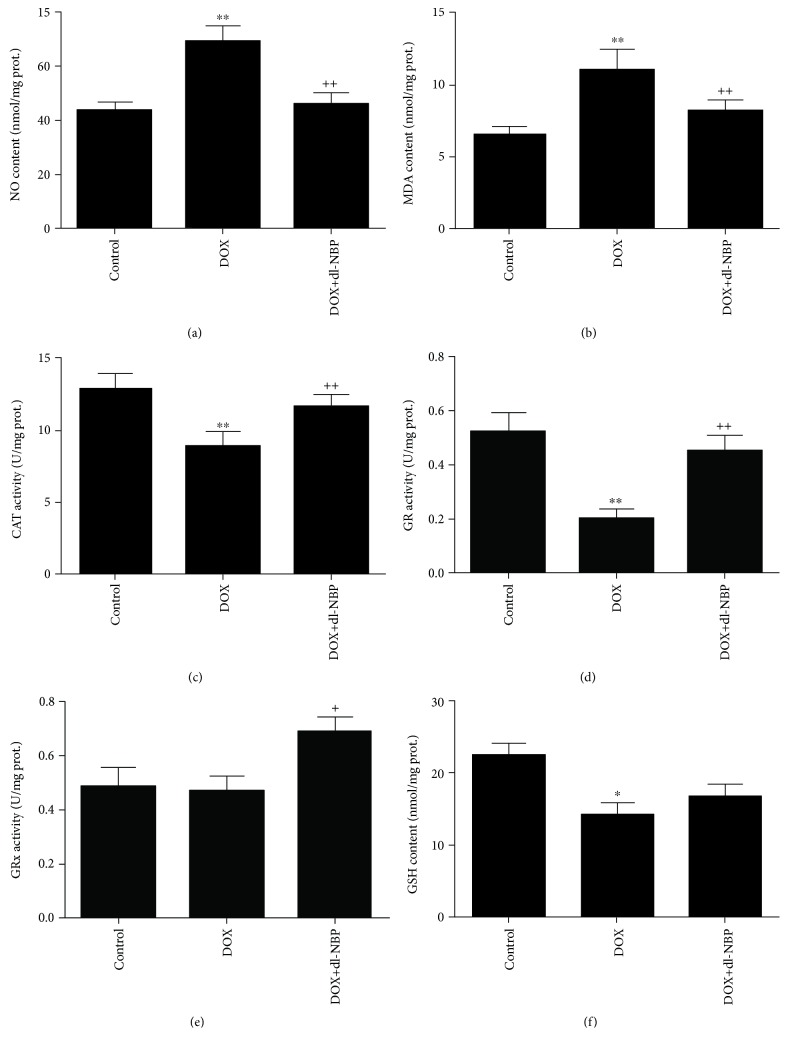
Effects of DOX and dl-NBP on oxidative stress markers in the hippocampus: NO content (a), MDA content (b), CAT activity (c), GR activity (d), GPx activity (e), and GSH content (f). Data are expressed as means ± SEM (*n* = 8). ^∗^ *p* < 0.05 and ^∗∗^ *p* < 0.01 compared to the control group. ^+^ *p* < 0.05 and ^++^ *p* < 0.01 compared to the DOX-injected group.

As shown in Figures 2(a) and 2(b), administration of DOX significantly increased levels of NO (*F*_2,21_ = 9.79; *p* < 0.01) and MDA (*F*_2,21_ = 8.52; *p* < 0.01) as compared to the normal control group, whereas treatment with dl-NBP significantly blocked the increasing of NO and MDA when compared to the DOX group. The parameter of CAT activity, GR activity, and GPx activity and the content of GSH are the major biomarkers of antioxidative defense system. The activities of CAT and GR were significantly decreased in rats treated with DOX as compared to the normal control group, whereas dl-NBP treatment caused a significant increase in the activity of CAT and GR as compared to DOX group (*p* < 0.01) (Figures 2(c) and 2(d)). The activity of GPx was not decreased after administration of DOX when compared with the control group, but the treatment with dl-NBP significantly increased GPx activity (Figure 2(e)) as compared to DOX-treated rats (*F*_2,21_ = 4.56; *p* < 0.05) (Figure 2(e)). DOX administration decreased the content of GSH significantly (*F*_2,21_ = 5.93; *p* < 0.05), whereas probably due to the fact that GSH was oxidized to neutralize DOX-induced excessive free radicals, slight, but nonsignificant increase of GSH concentration was observed in dl-NBP-treated rats compared with the DOX group (Figure 2(f)).

### 3.3. Effects of DOX and dl-NBP on Neuroinflammation Biomarkers

**Figure 3:**
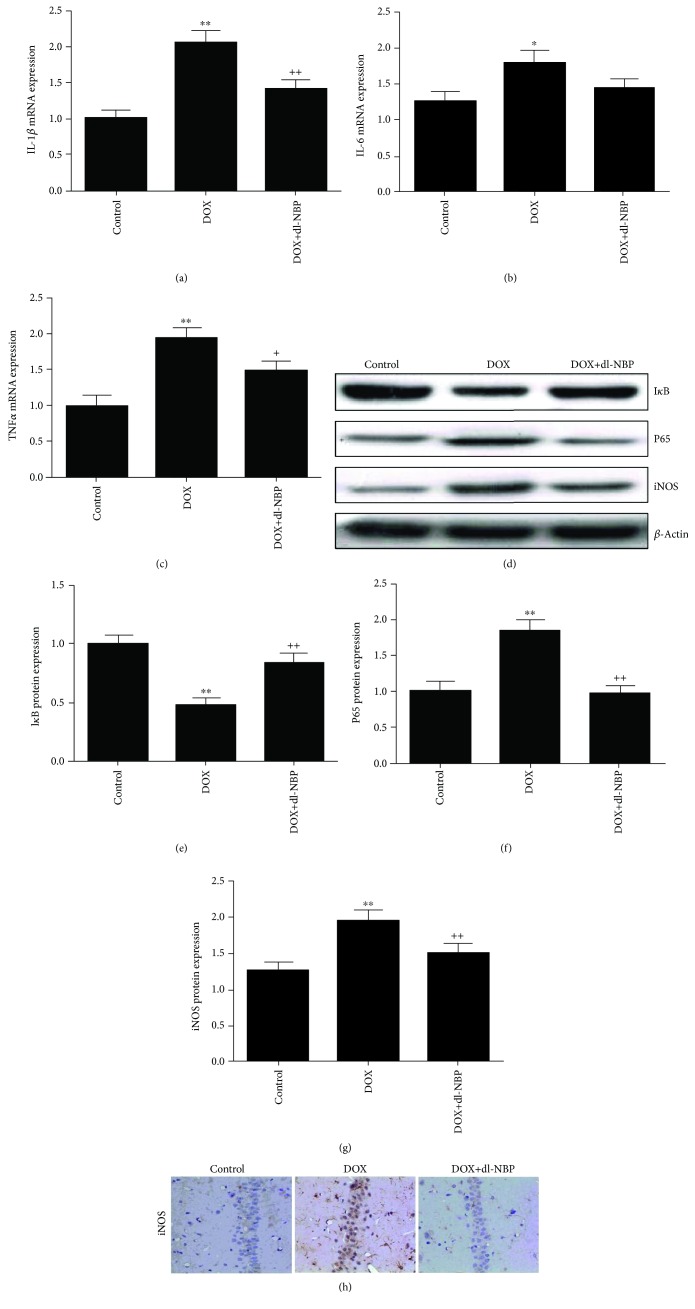
Effects of DOX and dl-NBP on neuroinflammation biomarkers: gene expression of IL-1*β* (a), IL-6 (b), and TNF-*α* (c); protein expression of I*κ*B (e), p65 (f), and iNOS (g); and immunohistochemical staining of iNOS (h). Data are expressed as means ± SEM (*n* = 8). ^∗^ *p* < 0.05 and ^∗∗^ *p* < 0.01 compared to the control group. ^+^ *p* < 0.05 and ^++^ *p* < 0.01 compared to the DOX-injected group.

The gene expressions of IL-1*β* (Figure 3(a), *F*_2,21_ = 13.32; *p* < 0.01), IL-6 (Figure 3(b), *F*_2,21_ = 3.87; *p* < 0.05), and TNF-*α* (Figure 3(c), *F*_2,21_ = 11.41; *p* < 0.01) were significantly increased in the DOX group. However, except IL-6, these elevated gene expressions were significantly attenuated by dl-NBP supplementation. The DOX + dl-NBP group showed significantly decreased gene expressions of IL-1*β* (Figure 3(a), *p* < 0.01) and TNF-*α* (Figure 3(c), *p* < 0.05) when compared to the rats in the DOX group. The DOX group showed a significant increase in protein expression of p65 (Figure 3(f), *F*_2,21_ = 13.46; *p* < 0.01) and iNOS (Figure 3(g), *F*_2,21_ = 7.02; *p* < 0.01) when compared to the control group. Consistent with the modulating effects of dl-NBP on the inflammatory cytokines, dl-NBP decreased the protein expression of p65 (Figure 3(f), *p* < 0.01) and iNOS (Figure 3(g), *p* < 0.01), and the immunohistochemical staining results of iNOS were in accordance with Western blot analysis (Figure 3(h)). The protein expression of I*κ*B was significantly suppressed in the DOX group (Figure 3(e), *F*_2,21_ = 14.65; *p* < 0.01) as compared to the control group; the treatment of dl-NBP significantly mitigated the reduction of I*κ*B protein level (Figure 3(e), *p* < 0.01) when compared to the rats treated with DOX alone.

### 3.4. Effects of DOX and dl-NBP on ER Stress

**Figure 4:**
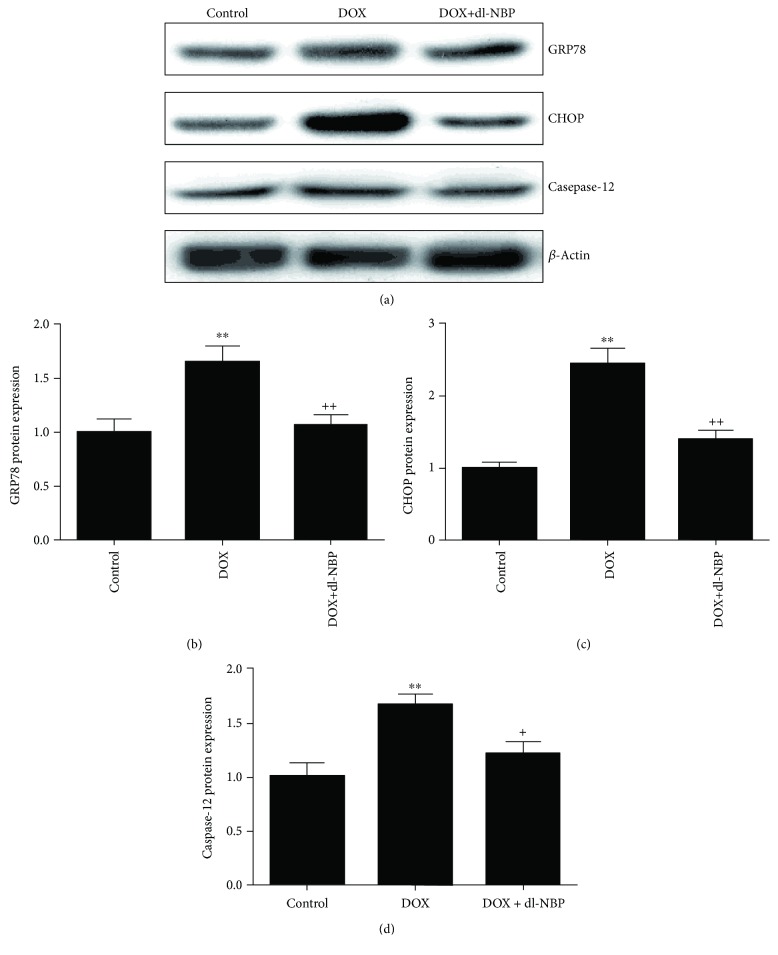
Effects of DOX and dl-NBP on ER stress. Protein expression of GRP78 (b), CHOP (c), and caspase-12 (d). Data are expressed as means ± SEM (*n* = 8). ^∗∗^ *p* < 0.01 compared to the control group. ^+^ *p* < 0.05 and ^++^ *p* < 0.01 compared to the DOX-injected group.

As the indicator of ER stress, the protein levels of GRP78, CHOP, and caspase-12 were monitored by Western blot to explore the mitigation effect of dl-NBP on DOX-induced hippocampcal ER stress [38]. As shown in Figure 4, the protein expressions of GRP78 (Figure 4(b), *F*_2,21_ = 7.85; *p* < 0.01), CHOP (Figure 4(c), *F*_2,21_ = 26.25; *p* < 0.01), and caspase-12 (Figure 4(d), *F*_2,21_ = 8.78; *p* < 0.01) were significantly increased after administration of DOX compared to the control group. Meanwhile the upregulation of GRP78 (Figure 4(b), *p* < 0.01), CHOP (Figure 4(c), *p* < 0.01), and caspase-12 (Figure 4(d), *p* < 0.05) protein expression were effectively inhibited by dl-NBP treatment.

### 3.5. Effects of DOX and dl-NBP on Histopathological Changes and Neural Apoptotic Markers

**Figure 5:**
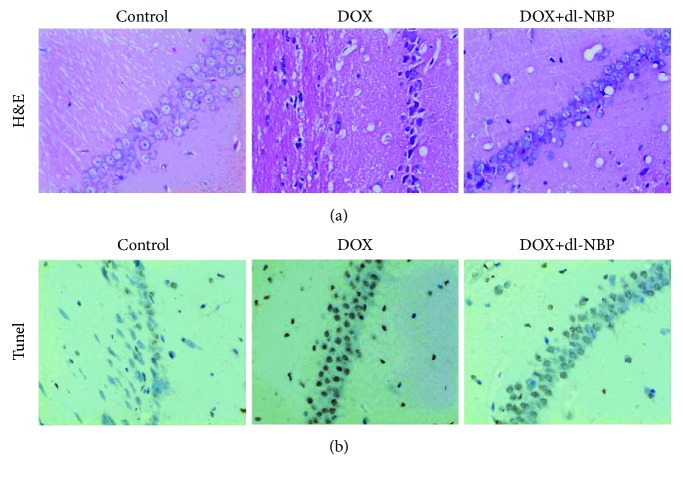
Effects of DOX and dl-NBP on histopathological changes and apoptotic markers. HE staining of different group (a) and TUNEL staining of different group (b).

Histopathological alternation in the hippocampus from different treated groups is presented in Figure 5(a). Compared with the normal histology in the control group, the hippocampus showed more frequent nuclear pyknosis in the DOX exposure group. In contrast, the treatment with dl-NBP was able to prevent the histopathological alternation evoked by DOX treatment. TUNEL test was used to assess apoptotic cells in the hippocampus of rats receiving different treatments. As revealed in Figure 5(b), fewer apoptotic cells were detected in the hippocampus of the normal treated control group. However, in the hippocampus of rats exposed to DOX, more TUNEL-positive cells were found as compared to the control group. Pretreatment with dl-NBP also markedly reduced TUNEL-positive cells, indicating the proapoptotic effects of DOX and antiapoptotic effects of dl-NBP in the hippocampus.

## 4. Discussion

Our study demonstrated the protective effect of dl-NBP against DOX-induced neurotoxicity in rats. We investigated the anxiety- and depression-like behaviors in rats exposed to DOX, and pretreatment with dl-NBP normalized behavioral changes in rats treated with DOX. Our study revealed that oxidative stress, neuroinflammation, ER stress, and cell death play a vital role in DOX-induced hippocampal damage. Moreover, we demonstrated that dl-NBP could partly alleviate these changes, suggesting the protective role of dl-NBP against DOX-induced neurotoxicity. Thus, our results provide a substantial support to those previously observed reports [42, 43] of neuroprotection by targeting oxidative stress, neuroinflammation, and ER stress cascade. dl-NBP might be an effective adjuvant therapy to prevent DOX-induced neurotoxic side effects in clinical practice.

As a chemotherapeutic agent, the long-term use of DOX tends to induce neurotoxicity and may cause neuropsychiatric diseases including anxiety and depression. Our previous study demonstrated that the underlying mechanism of behavioral changes following DOX treatment and the antidepressant-like and neuroprotective effects of *ω*-3 PUFAs were closely related to the oxidative stress, neuroinflammation, and apoptotic status in the brain tissues [27]. Similarly, our present study showed that the supplementation with dl-NBP effectively restored anxiety- and depression-like behaviors induced by DOX. Thus, we further evaluated various markers of oxidative stress, ER stress, inflammation, and apoptosis in different groups.

The oxidative stress, which is consisted of oxidation system and antioxidant system, can cause oxidative damage and promote inflammatory reactions. In our study, the levels of NO and MDA were significantly increased after exposure to DOX, showing that DOX increased oxidative damage. Meanwhile, the antioxidant enzymes, including CAT, GR, GPx, and GSH, were all significantly decreased in DOX-challenged rats. DOX increased oxidation stress system and decreased antioxidant system, and the emergence of the redox imbalance led to oxidative damage of nerve cells, which is accompanied with cognitive dysfunction, anxiety, and depression-like behaviors. Furthermore, we clearly demonstrated the capability of dl-NBP to downregulate the levels of NO and MDA and upregulate the levels of GSH and activities of CAT, GR, and GPx, which acts as an antioxidant thereby reducing oxidative stress-induced apoptosis.

Neuroinflammation plays a critical role in the pathogenesis of brain disorders [42, 44]. iNOS, which produces large amounts of NO, is active during the inflammatory process [45], activating proinflammatory mediators, such as TNF-*α* and NF-*κ*B, and subsequently induces brain neuroinflammation [43]. Proinflammatory cytokines such as TNF-*α*, IL-1*β*, IL-6, and iNOS have been demonstrated to play vital roles in inducing acute and chronic neurodegenerative disorders [42, 46]. We found that DOX provoked the generation of TNF-*α*, subsequently causing the activation of NF-*κ*B and iNOS and increasing the gene expression of IL-1*β* and IL-6 and protein expression of p65, indicating severe inflammatory conditions in the hippocampus, and these inflammatory may result in neural death and behavioral changes. The treatment with dl-NBP significantly suppressed the DOX-induced increase of TNF-*α*, IL-1*β*, IL-6, p65, and iNOS expression in brain tissues.

ER is an organelle which plays as a key role in protein folding. Various destructive stimuli and pathological conditions such as hypoglycemia, inflammation, oxidative stress, and hypoxia may impair the ER function and consequently lead to the induction of a self-protecting signaling pathway known as unfolded protein response (UPR) [47]. The connection to the UPR is induction of cytokines and inflammation that have been linked to depression. The UPR acts on proinflammatory cytokines such as IL-8, IL-1*β*, and TNF-*α*, and these cytokines have been found to be upregulated in patients with major depression [48]. ER stress and ER stress-evoked inflammation form a vicious cycle which ultimately leads to neuronal cell death through apoptosis. To evaluate ER stress after DOX exposure, protein levels of ER stress markers such as GRP78, CHOP, and caspase-12 were measured in the hippocampus in the current study. CHOP is a transcriptional factor, which could decrease expression of the antiapoptotic molecules and increase the expression of proapoptotic molecules to trigger apoptotic cell death [19, 49, 50]. GRP78 is a heat shock protein family chaperone transcriptional factor which plays a key role in the regulation of ER functioning [11]. Caspase-12 is an apoptosis-associated protein. Previous evidence has demonstrated that these ER stress-related proteins were also increased in the hippocampus of rat exposure to chronic unpredictable mild stress, a valid animal model of depression [38]. Our study found that dl-NBP alleviated the upregulation of CHOP, GRP-78, and caspase-12 in the hippocampus of rats exposed to DOX. Our results indicated that the potential antidepressant action of dl-NBP is endowed with its significant neuroprotective properties against DOX-induced hippocampal ER stress.

Our present study also found that DOX caused a significant increase in TUNEL-positive neurocytes, indicating severe DNA damage and neuronal death. Previous studies showed that DOX-induced neural apoptosis is closely related to depression [27]. Moreover, proinflammatory cytokines appear to contribute to depression-associated cell death through intrinsic apoptotic pathways, and neurotoxic free radicals are a second apoptosis-mediating factor associated with depressive disorder, suggesting that antioxidant and anti-inflammatory effects of dl-NBP could, in turn, indirectly contribute to its antiapoptotic effect. Although the study mainly focused on the neuroprotective effects of dl-NBP against DOX-induced neurotoxicity, it is important to note that the present study did not include dl-NBP-treated control animals, which is a major limitation of the study. Therefore, further studies are warranted to evaluate the baseline effect of the drug treatment to ensure its safety and efficacy.

## 5. Conclusion

In conclusion, our present study demonstrated that the DOX-induced behavioral anomalies might be the manifestations of oxidative stress, neuroinflammation, ER stress, and apoptosis in the hippocampus. The possible mechanisms under behavior-modulating and neuroprotective effects of dl-NBP are indicated to be at least partially associated with the antioxidant, anti-inflammatory, anti-ER stress, and antiapoptotic actions in the brain. Thus, our study provides a new potential treatment for brain damage induced by chemotherapeutic drugs and paves the way for further studies to investigate other mechanisms underlying the behavior modulating and neuroprotective effects of dl-NBP.

## Figures and Tables

**Table 1:** Primers used in real-time PCR analyses of mRNA expression.

Target gene		Primer sequences	Size (bp)
IL-1*β*	Forward	5′-AGGTCGTCATCATCCCACGAG-3′	119
	Reverse	5′-GCTGTGGCAGCTACCTATGTCTTG-3′	

IL-6	Forward	5′-CACAAGT CCGGAGAGGAGAC-3′	167
	Reverse	5′-ACAGTGCATCATCGCTGTTC-3′	

TNF-*α*	Forward	5′-GAGAGATTGGCTGCTGGAAC-3′	82
	Reverse	5′-TGGAGACCATGATGACCGTA-3′	

*β*-Actin	Forward	5′-CATCCTGCGTCTGGACCTGG-3′	116
	Reverse	5′-TAATGTCACGCACGATTTCC-3′	

## Data Availability

The data used to support the findings of this study are available from the corresponding author upon request.

## Supplementary Materials

Supplementary Materials Figure S1: the melting curve is shown as fluorescence versus temperature.
