# Supplementary material for: Neuroprotective Effects of dl-3-n-Butylphthalide against Doxorubicin-Induced Neuroinflammation, Oxidative Stress, Endoplasmic Reticulum Stress, and Behavioral Changes
Source: Oxid Med Cell Longev. 2018 Aug 16;2018:9125601. doi: 10.1155/2018/9125601 (PMC6116408; doi:10.1155/2018/9125601)
Supplement: Supplementary Materials — Figure S1: the melting curve is shown as fluorescence versus temperature. [file 9125601.f1.pdf]

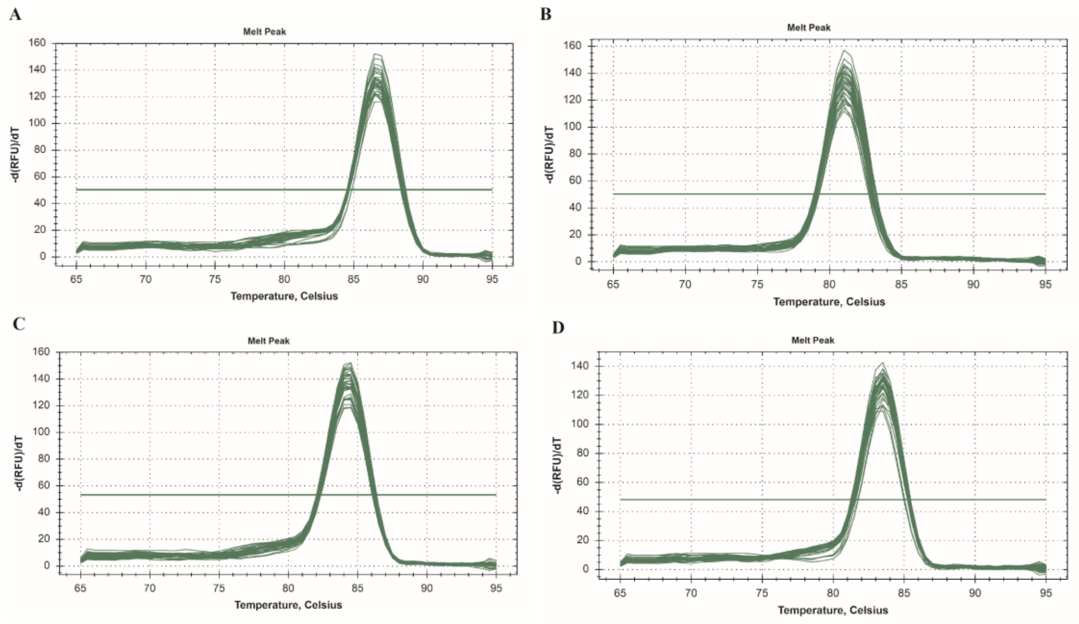

SUPPLEMENT FIGURE 1. The melting curve is shown as fluorescence (RFU) vs. temperature (T). When DNA melts, an abrupt decrease in fluorescence is seen in the form of a negative derivative of the melting curve, −d (RFU)/d T. Here, the melting temperature for β-actin (FIGURE 1A), IL-1β (FIGURE 1B), IL-6 (FIGURE 1C) and TNF-α (FIGURE 1D) was determined to be 87℃, 81.5℃, 84.5℃ and 84 ℃ respectively.
